# Patients with irritable bowel syndrome are more burdened by co-morbidity and worry about serious diseases than healthy controls- eight years follow-up of IBS patients in primary care

**DOI:** 10.1186/1471-2458-13-832

**Published:** 2013-09-11

**Authors:** Åshild Faresjö, Ewa Grodzinsky, Claes Hallert, Toomas Timpka

**Affiliations:** 1 Department of Medicine and Health, Community Medicine, Linköping University, Linköping, Sweden; 2 Unit of Research and development , County Council of Östergötland, Linköping, Department of Medicine and Health, Drug Research, Linkoping University, Linköping, Sweden; 3 Department of Social and Welfare Studies, Linköping University, Linköping, Sweden

**Keywords:** Functional gastrointestinal disorder, Co-morbidity, Case–control, Public health problem, Disease worry, Gender

## Abstract

**Background:**

Irritable Bowel Syndrome (IBS) is a hidden public health disease that affects up to 20% of the general population. Although co-morbidity can affect diagnose setting and treatment of the disease, there are few studies concerning diagnosed and registered co-morbidity for IBS patients in primary care. The aim of this study was to analyse co-morbidity among IBS-patients compared to age- and sex-matched controls from the general population using data from a county-wide computerized medical record system.

**Methods:**

IBS cases were recruited from three Swedish primary health care centres during a five-years period and controls from the same corresponding geographical areas. Co-morbidity data for IBS-patients and morbidity data for controls were derived from a population-based Health Care Register (HCR) covering all diagnoses in primary as well as hospital care in the region. Odds Ratios with 95% confidence intervals for morbidity in gastro-intestinal and non-gastrointestinal diagnoses for cases with irritable bowel syndrome compared to controls were calculated separately for each gender and diagnosis.

**Results:**

We identified more co-morbidity among IBS patients of both sexes, compared to matched controls in the general population. Patients with IBS were particularly more worried about having a serious disease than their control group. The risk among male IBS-cases to get this latter diagnose was three times higher compared to the male controls.

**Conclusions:**

In this population based case–control study, the analysis of diagnoses from the HCR revealed a broad spectrum of common co-morbidity and significantly more physician-recorded diagnoses among IBS-patients in comparisons to the control group.

## Background

Irritable bowel syndrome (IBS) is a common functional gastrointestinal disorder (FGD) which affects up to 20% of the general population, but can be considered as a “hidden” public health disease. IBS has been reported to be associated with a broad variety of psychological and physical symptoms and discomforts, as well as impaired quality of life and increased use of health care resources [1-6]. In this group of patients, a common reason for seeking health care is fear of severe gastrointestinal (GI) or other illness [7-9]. Adequate consideration of co-morbidity is of vital importance for accurate diagnosis of FGDs and choice of treatment for this patient group. Although FGDs are not life-threatening, exclusion of serious diseases, such as different types of gastrointestinal cancer, is a major clinical challenge. The clinical decision-making is therefore often difficult, in light of that psychological, social and biological factors all play a role, although the impact of each of these factors is likely to be different in different patients and may vary over time for the same person [5,6,10-12]. The treatments available today are beginning to address the multifactorial aetiology, and cover both pharmaceutical and non-pharmaceutical treatments, e.g. hypnotherapy and cognitive behavioural therapy [13,14]. Contrary to several of these findings, our recent study in Swedish primary care showed that IBS patients were not high consumers of health care and that most IBS patients consulted their general practitioner (GP) only for their abdominal problems [15,16]. Other studies have shown that approximately 50% of IBS patients from primary care and specialist clinics have at least one co-morbid symptom [17]. Hudson et.al reported that co-morbidity related to IBS includes fibromyalgia, migraine, chronic fatigue syndrome, major depression and panic disorder [18]. Patients with one or more co-morbid complaint tend to report more severe IBS symptoms, more mental complaints and more illness related absenteeism than patients without co-morbid disorders [17,19-22]. Psychosocial factors may also influence the health care seeking behaviours in addition to the irritable bowel syndrome itself. Anxiety and depression have been reported being more common in IBS patients than healthy controls. However, the causal relationship between IBS and psychosocial factors are still unknown [5,23].

Although co-morbidity can affect diagnose setting and treatment of IBS, there are few studies concerning diagnosed co-morbidity for IBS patients in primary care [24]. This study sets out to examine the 8-year prevalence of co-morbidity, morbidity, and worry about serious disease according to physician diagnoses among IBS-patients compared to controls from the general population using data from a regional Health Care Register (HCR) covering all levels of care. The research hypotheses are that IBS patients are more burdened by co-morbidity and disease worry than the general population.

## Methods

This present study is a part of a larger population-based case–control study [5,15,16]. Cases were patients from a Swedish primary health care (PHC) area receiving an IBS diagnose (ICD-10-P code K-58-p) according to Manning/Rome II diagnostic criteria [25] during a 5-year period (1997–2001). For all primarily identified IBS cases, the medical records were checked to ensure that there had not been any earlier IBS diagnosis. We found 115 cases with a prior confirmed diagnosis in the medical records (before 1997).Furthermore, seven IBS cases patients (three males and four females) died during the follow-up and four had a sheltered and non accessible medical record. Consequently, these cases were excluded. Controls were randomly selected from the census register for the population in the same geographical area using matching criteria for age and sex. Prior to the survey, a check was made to ensure that individuals in the control group did not have any registered IBS diagnosis at baseline. Data on co-morbidity, morbidity and worry about serious disease during an 8-year period for cases and controls were collected from a regional Health Care Register (HCR). Disease worry was defined by the corresponding ICD-10-P diagnose code Z71.1.

### Study population

The study was performed in Linköping (population 143.000), south-east of Sweden. The primary study population was defined by the geographical area covered by three urban PHC centres that provided PHC services to approximately 40.000 inhabitants. Before the investigation started, a pilot study was performed to develop a data registration form at one PHC centre. The medical records of fifty IBS cases with the code number K-58-p according to ICD-10-P were used for this purpose.

IBS cases were identified retrospectively from medical records at the three selected PHC centres for the period between 1/1 1997 and 31/12 2001. The medical records are not freely available for everyone, access is given after an ethical approval to researchers. Diagnosis, date of diagnosis, symptoms and data on number of health care visits, reason for consulting GP and demographic data were available in the medical records. In this case–control study, only IBS cases in working ages between 18–65 years were included, which resulted in N = 515 IBS cases in all. Through the local census population register, 4.500 sex-matched controls in the corresponding ages were randomly selected. The number of controls was chosen proportionally following the size of the actual population living in each of the three PHC areas (i.e. 2.100, 1.500 and 900 controls from the respective PHC areas). We found that 493 out of 515 patients and 2.773 out of 4.500 controls were registred in the HCR. The analysis is based on 493 patients and 2.773 controls.

### Data collection

To collect data on co-morbidity on 493 IBS cases and morbidity on 2773 controls, we used a regional HCR. This specifik database is not freely available for everyone, access is given after an ethical approval only to researchers from the university and employees from the county concil. This system is based on a unique personal code to birth date and gender of all inhabitants in the region. The database comprises diagnoses from primary care, hospital outpatient and hospital inpatient care was at the time of the study the only register of this type in Sweden [26]. ICD 10-codes were used for identification of morbidity in cases and controls during the period 1999–2007, two years after the first diagnosed cases (1997) and 6 years after the latest diagnosed cases (2001). All diagnostic codes were assigned by the GP or attending physician. The diagnoses were extracted from the HCR using a case-finding algorithm that retrospectively searched the register from 1/1/1999 to 31/12/2007.

### Statistical analysis

All data were stored in a common database and statistically analysed using the SPSS version 17.0 program (SPSS Inc., Chicago, IL, USA. 8-year prevalence of general and GI-specific morbidity was used as measure for the comparisons between cases and controls. 8-year morbidity prevalence was defined as having received specified diagnoses during the period 1999–2007. The Odds Ratios (OR) and 95% confidence intervals (CI) for co-morbidity was calculated separately for males and females and for IBS-patients and controls. In the HCR, the following case definition was applied: the first contact with health care services with different diagnoses during the period studied (1999–2007) was regarded as a case, the algorithm captured the cases (one case = one patient) regardless of whether the disorders of interest constituted the main or secondary diagnosis, and it also specified the health care level at which the patient was diagnosed.

### Ethical approval

The Ethical Committee at the Faculty of Health Sciences, Linköping University, Sweden, approved this study in 2002 and 2007 (Dnr M93-07).

## Results

In this study population, 72% of the IBS cases were female and more than 50% were below the age of 45 at baseline the controls were age and sex matched. See Table 1. The 8-year general morbidity was higher among IBS-patients in comparison to the control group. Also the prevalence of specific GI morbidity was higher among cases of both sexes compared to their control groups.

**Table 1:** IBS cases (n = 493) and controls (n = 2773) divided in sex and age

	**IBS cases**		**Controls**	
	**n**	**%**	**n**	**%**
**Sex:**				
Male	140	28.0	1292	46.5
Female	353	72.0	1481	53.4
**Age:**				
18 to 24	40	11.3	435	15.7
25 to 44	147	41.6	1171	42.2
45 to 64	166	47.0	1167	42.0

The ICD-diagnosis “worry about serious disease” Z71.1 was more common among IBS cases of both sexes compared to their controls; more than three times frequent among males OR = 3.30 (95% CI 1.93-5.66) and almost twice as common among females OR = 1.62 (95% CI 1.21-2.18), see Table 2.

**Table 2:** Eight-year prevalence of non-gastrointestinal morbidity in IBS-patients and controls

**Diagnosis**	**Female IBS cases N = 353**		**Female controls N = 1481**				**Male IBS cases N = 140**		**Male controls N = 1292**			
	**n**	**%**	**n**	**%**	**OR**	**CI 95%**	**n**	**%**	**n**	**%**	**OR**	**CI 95%**
Worry about serious disease	73	21.1	205	14.0	1.62	1.21–2.18**	20	14.3	62	5.1	3.30	1.93–5.66***
Cardiovascular complaints Stroke, chest pain UNS	180	51.1	288	19.4	4.31	3.37–5.51***	89	64.1	331	26.1	5.06	3.51–7.31***
Hypertension	90	25.5	226	15.3	1.90	1.44–2.51 **	36	26.0	232	18.1	1.58	1.05–2.37**
Diabetes ^1^	30	8.5	90	6.1	1.43	0.93–2.21	12	8.6	129	10	0.84	0.45–1.57
High Cholesterol	43	12.2	95	6.4	2.02	1.38–2.96 **	33	23.6	151	11.7	2.33	1.52–3.57***
Obesity	16	4.5	67	4.5	1.00	0.57–1.75	7	0.5	42	3.3	1.56	0.69–3.56
Thyroid diseases	52	15.0	133	8.9	1.75	1.24–2.47 **	5	3.6	55	4.3	0.83	0.33–2.11
Asthma/allergy	114	32.2	373	25.1	1.42	1.10–1.83 *	38	27.1	228	18.0	1.74	1.16–2.59*
Eczema	58	16.4	123	8.3	2.17	1.55–3.04***	19	13.6	86	6.6	2.20	1.29–3.74*
Obstructive lung disease	10	2.8	22	1.5	1.93	0.91– 4.12	8	5.7	12	1.0	6.46	2.60–16.10*
Arthritis	51	14.4	92	6.2	2.55	1.77–3.67***	22	15.7	79	6.1	2.86	1.72.4.72*
Musculoskeletal problems	104	29.5	263	18.0	1.93	1.48–2.52***	40	28.6	183	14.5	2.42	1.63–3.61***
Myalgia/rheumatism UNS	211	60.0	573	28.7	2.35	1.86–2.99***	65	46.4	345	27.0	2.38	1.67–3.39***
Pain and suffering UNS	48	14.1	120	8.1	1.78	1.25–2.55**	19	13.6	72	5.6	2.66	1.55–4.56**
Migraine/headache	142	40.2	310	21.0	2.54	1.99–3.25***	39	28.1	135	10.4	3.31	2.20–4.99***
Mental problems ^2^	213	60.0	275	19.1	6.67	5.19–8.56***	47	33.6	194	15.0	2.86	1.81–3.45***
Stress	68	19.3	244	16.5	1.20	0.90–1.63	19	14.0	97	7.5	1.93	1.14–3.27*
Tinnitus	15	4.2	44	3.0	1.45	0.80–2.64	5	3.6	38	3.0	1.22	0.47–3.20
Eating disorder	1	0.3	11	0.7	0.38	0.05–2.95	3	2.1	1	0.01	28.27	2.92–273.6***
Cancer	14	4.1	16	1.1	3.78	1.83–7.83***	1	0.7	5	0.4	1.85	0.22–15.96
Cold, flu/respiratory infections	56	16.1	121	8.2	2.12	1.51–2.98***	20	14.3	55	4.3	3.75	2.17–6.47***
Infection in the urinary tract	48	14.1	84	6.1	2.62	1.80–3.81***	16	11.4	28	2.2	5.82	3.08–11.06***
Disease in the kidney	11	3.1	6	0.4	7.91	2.90–21.53**	3	2.1	12	0.9	2.34	0.65–8.38
Gynaecological/pregnancy/ ^3^Urological/prostate problems^4^	19	5.4	64	4.3	1.26	0.74 –2.13	3	2.1	9	0.7	3.12	0.83–11.67
Fatigue and malaise	117	33.1	225	15.2	2.77	2.13–3.60***	31	22.1	101	8.0	3.35	2.14–5.25***
Psychosocial factors in daily life	5	1.4	11	0.7	1.85	0.87–3.92	2	1.4	10	0.8	1.85	0.21–4.66
Investigation of serious illness	16	4.5	19	2.0	3.65	1.86–7.18***	1	0.7	7	0.5	1.32	0.16–10.83
Miscellaneous diseases	185	52.4	362	24.4	3.40	2.68–4.33***	52	37.1	266	21.1	2.80	1.58–3.29***

**1.** Both insulin dependent and insulin independent diabetes. **2.** Includes anxiety, depression, sleeping problems, worries etc. **3**. Concerns women **4.** Concerns men. ***p = 0.001, **P = 0.01, *p = 0.05.

In general, females with an IBS diagnosis had more recorded co-morbidity during the 8-year period than male IBS-patients. In particular, females were to a larger extent than female controls diagnosed with psychiatric disorders (depression, anxiety and worries) and diagnoses related to musculoskeletal problems and pain (arthritis, myalgia/rheumatism UNS, migraine/headache, pain and suffering UNS). Thyroid hormone problems, asthma/allergy, hypertension, high cholesterol, kidney problems and infection in the urinary tract were also slightly more registered among females with an IBS diagnosis compared to females in the control group. This pattern was also seen concerning cancer and respiratory tract infections, as well as fatigue and malaise, see Table 2. Male cases showed almost the same pattern as the female did with small exceptions. Male IBS-patients had slightly more stress diagnoses compared to their control group and less cancer diagnoses.

Almost all IBS cases had additional unspecific FGD diagnoses registered in the HCR during the 8-year period. However, the female IBS cases were also diagnosed with more cancer in the abdominal tract than their control group as well as more inflammatory bowel disease. Male cases had on the other hand more haemorrhoids and rectal abscesses, lactose intolerance and celiac disease than their control group, see Table 3.

**Table 3:** Eight-year prevalence of gastrointestinal morbidity in IBS-patients and controls

**Diagnosis**	**Female IBS cases N = 353**		**Female controls N = 1481**				**Male IBS cases N = 140**		**Male controls N = 1292**			
	**n**	**%**	**n**	**%**	**OR**	**CI 95%**	**n**	**%**	**n**	**%**	**OR**	**CI 95%**
Reflux complaints (GERD)	73	21.1	77	5.5	4.75	3.37–6.71***	21	15.0	70	5.4	3.08	1.83–5.20***
Complaints with known ulcer	5	1.4	5	0.3	4.24	1.22–14.73*	4	2.9	11	1.0	3.42	1.08–10.90*
Functional dyspepsia	134	38.1	128	8.6	6.47	4.88–8.57***	53	35.7	92	7.1	7.94	5.32–11.88**
Inflammatory bowel disease	28	7.9	62	4.2	1.97	1.24–3.13***	6	4.3	41	3.2	1.37	0.57–3.28
Diverticulitis in the bowel	33	9.3	12	0.8	12.62	6.44–24.71 ***	10	3.6	55	4.3	9.86	4.03–24.13***
Haemorrhoids and rectal abscesses	25	7.1	67	4.5	1.61	1.00–2.59	22	15.7	58	4.5	3.97	2.35–6.71***
Liver diseases	3	0.8	5	0.3	2.53	0.60–10.65	0	0	17	1.3	–	–
Gallstone, gallbladder inflammation and other diseases in the gallbladder area	30	8.5	55	3.7	2.41	1.52–3.82**	8	5.7	20	1.5	3.85	1.67–8.92**
Lactose intolerance	1	0.3	9	0.6	0.46	0.06–3.68	2	1.4	2	0.2	9.35	1.31–66.88*
Coeliac disease	4	1.1	13	0.9	1.39	0.42–3.99	3	2.1	4	0.3	7.05	1.56–31.83*
Irritable bowel syndrome ^1^	30	8.5	8	0.5	–	–	6	4.3	5	0.4	–	–
Gastroenteritis	4	1.1	5	0.3	3.38	0.90–12.66	1	0.7	5	0.4	1.85	0.21–15.96
Cancer in the abdominal tract ^2^	8	2.3	5	0.3	6.84	2.26–21.05 ***	2	1.4	6	0.5	2.55	0.62–15.54
Benign tumours in the abdominal tracts	18	5.1	9	0.6	8.79	3.92–19.73 ***	4	2.9	10	0.8	3.77	1.17–12.18*
Miscellaneous gastrointestinal complaints	6	1.7	18	1.2	1.40	0.55–3.57	6	4.3	26	2.0	2.18	0.88–5.39

**1.** For IBS cases, this is a renewed diagnose set after 2001, for the controls it is new diagnose, set after 2001. **2.** Includes cancer diagnose in colon, ventricle and rectum. ***p = 0.001, **p = 0.01, *p = 0.05.

## Discussion

It is well known that broad spectrums of diagnostic procedures are needed to exclude other conditions when IBS is suspected, due to an overlap between different GI diseases. Studies have also previously reported GI and other co-morbidity among IBS cases; but many of these studies have been performed among IBS health care seekers [27-31] and only a few have been based on a population-based design [23,24]. The main findings of this study are that IBS cases more often receive the diagnosis “worry about having a serious disease” (ICD-10 Z71.1) than their control group. This particular ICD-diagnosis is established and used when an individual consult his physician claiming fears of having a serious disease, but after doctors’ examination no further diagnose could be set. The risk among male IBS-cases to get this diagnosis was three times higher compared to the male controls. As also reported from previous studies, IBS cases of both sexes had in general more diagnoses recorded in the HCR compared to matched controls in the general population. However, the design of the study does not permit us to draw conclusions about whether a recorded co-morbidity was a cause or consequence of IBS. Neither did the chosen study design allow us to further analyse the findings that IBS patient seemed to be more worried of having a serious disease, than their controls. This was an additional finding in this study and more research is warranted on this issue.

Non-GI symptoms form one important part of IBS patients’ complaint panorama, which affects diagnose setting and treatment of the disease [29,32]. Our previous results pointed out that non-GI co-morbidity affected the use of heath care as well as a strong predictor for follow-up visit to the GP among IBS patients [16]. Higher frequencies of anxiety and depression as well as sleeping problems, have previously been recognized as factors associated with IBS diagnosis [23,33-39]. IBS and anxiety disorders are reactive to stress and are likely to involve serotonergic disturbance, which include anticipatory worries and avoidance behaviours that impair quality of life as well as functioning in everyday life [5,40-42]. This complex form of co-morbidity probably involve psychological and physiological processes that trigger each other and form a spiral between the two types of disorders, completely independent of sex. Fatigue and malaise could be a result of dealing with both psychological and physiological symptoms and the presence of this particular diagnosis was more frequent among the IBS cases. Fatigue has also been identified as the most common somatic symptom associated with IBS in India [30].

IBS patients were found to suffer more frequently from headache and migraine than their controls, which other studies confirm [29,43,44]. This might due to severe IBS symptoms or the other way around, i.e. that these particular co-morbidities contribute to worsen the IBS symptoms through unknown mechanisms. The brain-gut axis could be involved as well as neuroendocrine and neuroimmune interactions [43,45,46]. Fibromyalgia is the most frequently investigated co-morbidity of IBS [29,47,48]. In this study, fibromyalgia was recognized as co-morbidity only among females. Very few had a registered diagnosis of fibromyalgia, but myalgia/rheumatism UNS occurred more frequently among all IBS cases compared to the control group. Additionally, musculoskeletal problems, arthritis and pain and suffering UNS were significantly more common among IBS cases of both sexes. One could hypothesize that all these pain-related complaints might lead to a larger consumption of analgesics resulting in damage in the mucosa in the GI canal which might worsen the existing symptoms or new GI symptoms will occur. Treatment of, for instance, arthritis often includes a short-term use of non-steroidal anti-inflammatory drugs (NSAID) [49]. Previous results from the present research program showed that IBS cases of both sexes consumed significantly more analgesics than their controls did [50]. Registered diagnoses of thyroid disease, asthma, allergy, infections in the urinary tract as well as cardiovascular problems were more frequent among the IBS cases. The majority of co-morbidity diagnoses in this study were set after the initial IBS diagnosis. One can speculate if IBS diagnosis is a marker for other diseases or simply a reason for follow-up visits to health care were these co-morbidity are discovered. These observations of co-morbidity in several extraintestinal organ systems might raise the suspicion that the physician’s specialisation influences the diagnosis of morbidity in relation to IBS. A hypothesis is that medical subspecialisation is responsible for an artificial separation of one and the same disease, i.e. a specialist in gastroenterology will use the diagnosis IBS, a rheumatologist fibromyalgia, etc. [51]. Therefore it is important for physicians to exclude other organic diseases and refer IBS patients with somatic co-morbid symptoms to specialists within that particular area. In other words, it is important to arrive at the correct diagnose in order to be able to provide an adequate treatment.

Various GI co-morbidity were frequent more common in IBS cases such as functional dyspepsia (FD) and reflux complaints (GERD), for both sexes. An overlap between IBS-typical and FD typical symptoms can be found in individuals with FGD [32]. Moreover, the abdominal complaints in individuals with a FGD often change over time resulting in variations of predominant symptoms in almost the majority of the patients during a year. This circumstance may explain the high frequency of unspecific different FGD diagnoses within the patient group almost every case had two or more FGD-UNS diagnosis previous to the IBS diagnosis. One hypothesis is that the motility disturbance of the gastrointestinal tract is involved in the complex pathophysiology of GERD, FD and IBS might result from a common neuromuscular dysfunction [52-54]. Other, GI diagnoses seen were inflammatory bowel disease, diverticulitis, gallstone, gallbladder inflammation and other diseases in the gallbladder area as well as haemorrhoids and rectal abscesses. The latter was the only diagnosis where a gender difference could be seen, with more males suffering from these complaints. In consideration of the Rome criteria, an organic disease must be excluded before the diagnosis of IBS is set. However, due to the relapsing character of ulcerative colitis and Chron’s disease, many patients have long-standing remission without any sign of active inflammation and in these patients an increased prevalence of IBS-like symptoms have been found [55]. One hypothesis is that IBS subgroups with post-infectious IBS might share pathophysiological mechanism with IBD patients in remission who suffer from IBS-like symptoms, which might explain the frequency of IBD diagnoses among the cases [55-57]. Although, all IBD diagnosis was set after the initial IBS diagnose, otherwise these cases would have been excluded from the study at baseline. There seems to be some increase in cancers in the GI-tract for female IBS-cases in this study, one must be very careful when interpret such associations, because it is few cases and consist of three GI-cancer diagnoses. Benign tumours in the abdominal tract were also seen more frequently among cases of both sexes compared to their control group.

One major limitation of this case–control study is that the design does not permit to draw conclusions about whether a recorded co-morbidity is causally related to IBS. However, the study also has several strengths. One strength is that it is based on computerized Health Care Records (HCR) for all inhabitants linked by birth date and sex. The same personal code is used for all visits and diagnoses in Health Care registers (HCR). An individual can thus be followed retrospectively or prospectively through the health care system using this personal code. The health care institution where the patient was diagnosed represents all health care levels—primary care, outpatient hospital care, and/or inpatient hospital care which gives a more complete panorama of the health care. Other strengths of this study are that our inclusion method gains reliability through being very general and covering a span of several years and that the number of included patients is high tending to level out possible misclassification within the groups defined as patients. However, a study of the quality and content of the Swedish Hospital Discharge Register indicates 95% coverage of main diagnostic codes in inpatient care in this region in 1986 and 98% in 2002 [58]. Validation of HCR and other administrative data has shown high specificity in registers covering all types of health care [26,59]. But a constraint for register data is that misclassifications do occur, including cases that are not recorded because they are overlooked or given incorrect clinical codes. ICD code registration could vary between physicians and health care centers and also between diagnoses [60]. Nevertheless it can be assumed that the data used in this study based on a HCR has high reliability, the majority of the patients and controls had registred the same diagnosis code in HCR several times during the study period, so we could be certain of the accuracy of the diagnose settning.

Moreover, the study was based on a population-based case–control design [61]. Further, prior to the survey, a check was made to ensure that individuals in the control group did not have any registered IBS diagnose during the period studied. We calculated the number of controls to this study in accordance with the epidemiological well-established principle; when having a case–control study, every identified case should at least have two or preferably at least three controls each from the general population. Another possible limitation in using IBS diagnoses from medical records, as we have in this study, is the dependence on the GP’s ability to make the correct diagnosis. However, studies have shown that GPs rarely misdiagnose patients, and in particular not IBS [62-64]. There could, on the contrary, rather be a tendency to under-diagnosing of these complaints in primary care. Medical records in primary care in Sweden are generally regarded as a reliable source of such kinds of data collection since the PHC centres have an overall responsibility for the PHC in a catchment area, and therefore are required to regularly report morbidity patterns based on structured diagnosis. This weakness is the same in the case of most clinical research utilizing data from more than one health care provider [65,66].

## Conclusions

IBS patients seem to worry about serious diseases more than controls in the general population. They also seem to be burdened with more physician-diagnosed co-morbidity compared to age- and sex-matched controls in the general population. The casual direction of this co-morbidity needs to be further investigated.

## Abbreviations

CI: Confidence intervals; FD: Functional dyspepsia; FGD: Functional gastrointestinal disorder; GERD: Gastroesophagel reflux disease; GI: Gastrointestinal; GP: General practitioner; HCR: Health care register; IBD: Inflammatory bowel disease; IBS: Irritable bowel syndrome; ICD: International classifications of diseases; NSAID: Non-steroidal anti-inflammatory drugs; OR: Odds ratio; PHC: Primary health care; SPSS: Statistical package for the social sciences; UNS: Unspecific.

## Competing interests

The authors declare that they have no competing interests.

## Authors’ contributions

ÅF, EG, TT participated in the study design and coordination and completed the data collection. ÅF, EG ,TT, CH drafted the manuscript as well as analysis and interpretation of data, read and approved the final manuscript.

## Pre-publication history

The pre-publication history for this paper can be accessed here:

http://www.biomedcentral.com/1471-2458/13/832/prepub
